# Peer review: tips from field experts for junior reviewers

**DOI:** 10.1186/s12916-015-0512-3

**Published:** 2015-11-02

**Authors:** Sabina Alam, Jigisha Patel

**Affiliations:** BioMed Central Ltd, 236 Gray’s Inn Road, London, WC1X 8HB UK

**Keywords:** Junior reviewers, Peer review, Training

## Abstract

This editorial introduces a series of tutorials by experts, who provide tips and advice for junior reviewers on how to conduct peer review based on specific study designs. The aim of these articles is to provide an easy-to-use, quick reference for those who are seeking more guidance on how to peer review biomedical research papers. Unlike previous tips and guides on peer review, this series is the first to provide advice from experts for those in their specific fields.

## Editorial

As a general medical journal, *BMC Medicine* receives submissions from a wide variety of biomedical fields, using many different types of study design. We select reviewers on the basis of their reputation and expertise in relevant subject areas and methodologies; but this process makes the assumption that using these criteria results in good peer review skills.

Although untested, this is a widespread belief and, in the absence of any better way of assessing peer reviewer skills, it seems to work. Reviewer reports ultimately have the potential to influence what gets published. However, with the ever-increasing ‘burden’ of scholarly peer review that is placed upon the research community to meet the needs of journals and authors [1], these criteria (reputation and expertise) focuses this burden on a comparatively small number of people.

To address this burden, it is inevitable that the less experienced will be called upon to peer review more frequently, as the pool of ‘experienced’ peer reviewers runs dry. However, this presents a problem as there is a lack of formal training in peer review. This is often a skill learnt ‘on the job’ and dependent on having supportive mentors who might actually be able to offer advice.

The quality of peer review is every bit as important as peer review itself. However, a significant proportion of reviewers feel that guidance and formal training in peer review is needed [2]. Some junior reviewers point out that they are not entirely clear about what is required of them when they accept an invitation to review [3]. Also, as feedback on reviews is very rarely offered [4], there have been various initiatives about how to address this [5].

There is a need to provide training, and not just to support the less experienced. There are also advantages to standardizing and specializing peer review to some extent to better define what it is for. One of the more recent proposals includes journal editors offering training and recognition for reviewers, and providing readers with a way to identify individual published articles that have been assessed by ‘trained’ reviewers [6].

With these ideals in mind, we have undertaken an initiative to provide training for peer review. Following a series of blogs [7–9] on the generalities of how to peer review, we have commissioned a series of ‘specialist’ how to peer review tutorials for a new article collection in *BMC Medicine*, ‘*Peer review: tips for junior reviewers*’. The aim of these tutorials is to provide a source of quick reference on how to perform a critical peer review based on different study designs.

To write these articles, we invited experts who regularly review for us and other journals. Many are from our editorial board and so are also experienced in guiding editorial decisions on the basis of reviewer reports.

Peer reviewers guide editors to identify which papers can be published, and a key role is in identifying flaws in methodology and conclusions that are not backed by data. Reviewers are expected to tease apart ‘fatal flaws’ from potentially addressable flaws. In their tutorial, Del Mar and Hoffman provide examples of what can be considered to be a major flaw in a randomized controlled trial (RCT) [10]. Key areas to assess include risk of bias and, for RCTs specifically, Del Mar and Hoffman suggest that reviewers can use the mnemonic RAMbo (Randomized Attrition Measurement by blinded assessors or objective measures) as a useful reminder to assess key types of potential bias.

Critical peer review ensures appropriate reporting of research, which in turn facilitates reproducibility. With this in mind, reporting guidelines that exist for many different types of study designs [11] are just as useful for reviewers as they are for authors. As an expert in systematic reviews and meta-analyses, Moher points out that reporting guidelines are particularly useful for reviewers as several options exist for the different types of systematic review designs [12]. A thorough review will discuss different parts of methodology used in a single study. Moher stresses the importance of discussing the systematic review and meta-analysis parts separately, as this ensures that the different statistical requirements in the two different types of analysis have been assessed.

Of course, it may not always be possible for a subject reviewer to assess all the statistical analysis in a given manuscript, especially as study designs and statistical methods undergo developments and updates to fit the needs of researchers. In their tutorial, Greenwood and Freeman provide guidance for non-statistical reviewers explaining why it is also important for subject reviewers to be aware of when they should recommend to the editors that a given manuscript should undergo review by a statistical expert. To aid this, they provide a list of common statistical issues that may act as a prompt for the subject reviewer [13].

We will continue to add contributions from invited experts in various fields with the aim of shaping this article collection to provide a useful resource for reviewers across multiple disciplines. As with all of our journal content, these articles are freely available, and we hope that reviewers will find the advice to be constructive and useful.
